# Mechanistic study on integrated water-fertilizer management to alleviate Na^+^ toxicity and enhance salt tolerance and yield of pakchoi under salt stress

**DOI:** 10.7717/peerj.20431

**Published:** 2025-12-18

**Authors:** Jin Li, Hongcheng Li, Zizheng Li, Huirong Su, Tingting Duan, Zhong Lin, Yinling Zhu, Xiaoli Chen, Xianmin Wang

**Affiliations:** 1 Guangdong Ocean University, Zhanjiang, Guangdong, China; 2 Zhanjiang Agricultural Technology Extension Center, Zhanjiang, Guangdong, China

**Keywords:** Pakchoi, Salt stress, Integrated water-fertilizer management, Ion homeostasis and Na^+^ toxicity, Photosynthetic performance and ROS defense

## Abstract

The cultivation of salt-tolerant pakchoi is a promising strategy for utilizing coastal saline soils. However, supporting agronomic technologies are remain underdeveloped. This study investigates how coordinated water-fertilizer regulation mitigates sodium (Na^+^) toxicity under salt stress, thereby enhancing salt tolerance and yield in pakchoi and providing a scientific basis for optimizing agronomic management. A pot experiment was conducted with three irrigation methods (conventional, drip, and mulched drip) and three fertilizer types (conventional, controlled-release, and mixed), under soil salt stress adjusted by sodium chloride (NaCl) to 0.15% salinity by weight. The results showed that MP treatment (mulched drip irrigation + mixed fertilizer) can significantly improve yield performance, with fresh and dry weight increasing by 39% and 42% respectively, and enhancing ion balance by increasing potassium (K^+^) and calcium (Ca^2+^) and reducing Na^+^. In addition, MP enhanced antioxidant defense by increasing superoxide dismutase (SOD), catalase (CAT), peroxidase (POD), and ascorbate peroxidase (APX) activities and reducing oxidative damage. The treatment also maintains better water status and photosynthetic efficiency. MP treatment can improve growth, physiology, and stress tolerance. This integrated approach represents a novel water-fertilizer strategy with high application potential for sustainable vegetable production in saline regions.

## Introduction

Pakchoi (*Brassica rapa* L. ssp. *chinensis*) belongs to the Brassicaceae family and is native to China. It is commonly known as Chinese cabbage. It is an important leafy vegetable crop in China and has been extensively cultivated in Europe, Americas, Japan, and Southeast Asia, gradually becoming a globally recognized vegetable (Yu et al., 2022). Soil salinization is the main limiting factor for greenhouse vegetable production (Xu et al., 2018), and one of the most serious agricultural issues worldwide (Liang et al., 2018). About 950 million hectares of land are affected globally, including nearly 100 million hectares in China (Li et al., 2022a). In Guangdong, saline-alkali land covers nearly 200,000 hectares, with Zhanjiang accounting for about half (Luo, 2012). Although over 50% of arable land is expected to become saline alkali land by 2050 (Li et al., 2022b), this study focuses on local agricultural impacts rather than providing detailed global predictions, in order to better address the specific research gaps.

Coastal soil salinization typically occurs due to rapid soil moisture evaporation, leading to the gradual accumulation of sodium chloride (NaCl) in the soil (Khalifa et al., 2016). Under NaCl stress, plant growth and development are typically slowed; metabolic capacity is inhibited and severe wilting may occur, potentially leading to plant death (Parida & Das, 2005). Recent studies have revealed that post-transcriptional modifications, particularly N6-methyladenosine (m6A), play a critical role in regulating stress-responsive gene expression, providing new mechanistic insights into plant salt tolerance (Shan et al., 2025).

Pakchoi, as a nitrogen-loving leafy vegetable, is particularly vulnerable to salinity, where Na^+^ accumulation interferes with nutrient uptake, damages cell membranes and reduces yield (Liu et al., 2017; Piao, Zhao & Eun, 2020). Irrigation and fertilization are essential for supplying water and nutrients. Their mismanagement can paradoxically increase root-zone salt accumulation and intensify sodium (Na^+^) toxicity (Lima et al., 2020). Film mulched irrigation can reduce evaporation and salt accumulation (Lima et al., 2020), while controlled-release fertilizers can improve nitrogen efficiency and reduce soil salt accumulation (Zhang et al., 2016). Ion uptake, especially potassium (K^+^) and calcium (Ca^2+^), can inhibit Na^+^ entry and alleviate ion imbalance (Zhang et al., 2018). Since these advances, studies on pakchoi in coastal saline soils have mainly focused on breeding salt-tolerant varieties, with limited attention to integrated water-fertilizer management (Lima et al., 2019).

This study aims to investigate and evaluate the effects of different fertilization regimes (conventional, controlled-release, and mixed) and irrigation methods (surface irrigation, drip irrigation, and plastic-film mulched drip irrigation) under salt stress (0.15% NaCl, designated as soil salinity by weight). Evaluation includes soil electrical conductivity (EC), ion balance, nutrient contents, leaf water potential, osmolytes, ROS, MDA, electrolyte leakage, antioxidant enzyme activities, chlorophyll content, photosynthetic parameters, and growth and yield (Ibrahimova et al., 2021; Ibrahimova et al., 2025). This approach aims to clarify how integrated water-fertilizer management alleviates Na^+^ toxicity in pakchoi, providing a scientific support for optimizing production in coastal saline-alkali land.

## Materials and Methods

### Experimental materials

Test soil: Soil samples were taken from the cultivation layer (0–20 cm) of the breeding base in Guangdong Ocean University (N21°8′31″, E110°18′23″). Soil characteristics were pH 6.45, EC 670 µS/cm, salinity 0.01%, bulk density 1.20 g/cm^3^, organic matter content 14.25 g/kg, available nitrogen 65.57 mg/kg, available phosphorus 10.81 mg/kg, available potassium 58.37 mg/kg, water-soluble calcium 3.43 mg/kg, and water-soluble magnesium 2.28 mg/kg. After air-drying, these samples were crushed and sieved through a 2.00 mm mesh. Then five g NaCl was added and mixed evenly. After that, the soil was placed in pots (with an inner diameter of 21 cm, a height of 20 cm, no holes at the bottom), with each pot containing 3.8 kg soil. After adjustment, the measured soil salinity was 0.15%.

Test crop: Improved pakchoi (*Brassica rapa* L. ssp. *chinensis*) seeds of the “Jinpin 28” (Fujian, China) variety were purchased from Fujian Jinpin Agricultural Science and Technology Co., Ltd. This variety is known to thrive in soils with a total salt content of up to 0.3%. Its root system mainly extends within 10 cm of the top of the soil. The growth includes seedling stage (20 days), rosette stage (20 days), flowering stage (15 days), and fruiting stage (15 days). Due to its classification as a leafy vegetable, it is typically harvested during the later stages of the rosette period. The seeds were germinated in a cultivation box until they had two leaves and one heart. Even seedlings were selected and transplanted into experimental pots.

Test fertilizers: Fertilizers were provided by the Research Center of Environmentally Friendly Fertilizer Engineering Technology in Guangdong. Fertilizers included soybean oil coated compound controlled-release fertilizer (N:P_2_O_5_:K_2_O = 14:14:14, effective for 1–2 months), conventional urea (*N* ≥ 46.0%), monoammonium phosphate (N:P_2_O_5_:K_2_O = 12:61:0), and potassium chloride (K_2_O ≥ 60%). All fertilizers were evenly mixed into the soil as base fertilizer.

### Experimental design

The experiment was conducted in a glass greenhouse at the Guangdong Ocean University Agricultural Biotechnology Research Institute from October 23 to December 29, 2021. A two-factor split-plot design was employed. The main factor consisted of different irrigation methods, including conventional irrigation (I), trickle irrigation (T), and film mulched trickle irrigation (P). The sub-factor involved the application of different fertilizers are conventional fertilizer (F), controlled-release fertilizer (C), and mixed fertilizer (M) with F and C. In addition, a control treatment (CK) was established using conventional fertilizer (F) and conventional irrigation (I) without NaCl. There was a total of ten treatments (as shown in Table 1), each with five replicate pots containing three pakchoi plants.

**Table 1:** The design of experiment.

Treatments	Irrigation methods	Fertilizer types	With or without NaCl
FI	Conventional irrigation	Conventional fertilizer	With
FT	Trickle irrigation	Conventional fertilizer	With
FP	Film mulched trickle irrigation	Conventional fertilizer	With
MI	Conventional irrigation	Mixed fertilizer	With
MT	Trickle irrigation	Mixed fertilizer	With
MP	Film mulched trickle irrigation	Mixed fertilizer	With
CI	Conventional irrigation	Controlled-release fertilizer	With
CT	Trickle irrigation	Controlled-release fertilizer	With
CP	Film mulched trickle irrigation	Controlled-release fertilizer	With
CK	Conventional irrigation	Conventional fertilizer	Without

**Notes.**

MP (mixed fertilizer+film mulched trickle irrigation); DW (dry weight); FW (fresh weight); FI (conventional fertilizer+conventional irrigation); FT (conventional fertilizer+trickle irrigation); FP (conventional fertilizer+film mulched trickle irrigation); MI (mixed fertilizer+conventional irrigation); MT (mixed fertilizer+trickle irrigation); CI (controlled-release fertilizer+conventional irrigation); CT (controlled-release fertilizer+trickle irrigation); CP (controlled-release fertilizer+film mulched trickle irrigation); CK (control treatment).

### Experimental methods

Fertilization method: Nitrogen, phosphorus, and potassium fertilizers were evenly mixed with the soil as base fertilizer, with each treatment receiving 0.2 g N/pot, 0.2 g P_2_O_5_/pot, and 0.2 g K_2_O/pot. In the mixed fertilizer (M) treatment, it had a mixture of 50% NPK nutrients from the conventional fertilizer (F) and 50% from the controlled-release fertilizer (C).

Irrigation methods: For irrigation treatment, a graduated cylinder was used to evenly and quantitatively water the surface of potted plants. In trickle irrigation treatment, the water was measured using a graduated cylinder and added to the drip emitter with a constant dripping rate of 30 ml/h for targeted irrigation. For film mulched trickle irrigation treatment, soil in the pots was covered with black plastic film, and holes on the plastic film of the drip emitter outlets were made for local drip irrigation. Before the experiment, the field capacity of base soil was determined (26%), and each pot was supplied with an equal amount of water equivalent to 80% of the field capacity (790 ml). Subsequently, water was added daily between 6:00 PM and 7:00 PM to maintain the soil moisture content at 80% of the field capacity.

Collection of soil and plant samples: The experiment for transplanting pakchoi seedlings was conducted on October 23, 2021. On November 13, 2021, soil samples were collected for the first time during the seedling stage. Soil samples were taken from the middle of each pot, as well as from the upper layer (0–10 cm, root zone) and the lower layer (10–20 cm). A soil core was collected from each pot, and this process was repeated five times for each treatment (five pots per treatment). The samples were sieved through a two mm mesh to remove stones and plant roots, and then air-dried for analysis. On December 4, 2021, the same procedure was repeated during the later stage of the rosette stage of Pakchoi. Plant samples were harvested and washed with deionized water, blotted dry with absorbent paper to determine fresh weight. A small number of fresh leaves was taken to measure physiological index. The remaining plant samples were oven-dried at 75 °C to a constant weight and then ground for further analysis.

### Soil and plant sample measurements

Soil electrical conductivity (EC) was determined using a soil salinity meter (Germany, STEP PNT300 model) at a 1:1 soil-to-water ratio. Plant heights were measured using a ruler from the ground to the tip of the uppermost leaf in the main stem axis on days 7 (early seedling stage), 17 (late seedling stage), 27 (early rosette stage), and 37 (late rosette stage) after transplanting.

On the harvest day, functional leaves were sampled for physicochemical analysis. To minimize the influence of diurnal variations, all physiological measurements (such as photosynthetic parameters, antioxidant enzyme activity, and osmolytes) were conducted on fully expanded functional leaves at a consistent time of the day from 9:00 to 11:00 a.m. under stable light and temperature conditions. Leaf water potential was measured using the pressure chamber method with a PMS Model 1,000 instrument (USA), with fresh samples collected and measured in the early morning (Turner, 1988). Chlorophyll a, chlorophyll b, and total chlorophyll contents were extracted with 80% acetone and calculated based on absorbance values measured at 663 nm and 645 nm using a spectrophotometer (Lichtenthaler & Wellburn, 1983). On clear and windless days from 9:00 to 11:00 a.m., fully expanded functional leaves from the middle part of pakchoi plants were selected for measurement. A portable photosynthesis system (LI-6400XT, LI-COR Inc., Bourne, MA, USA) was used to determine key photosynthetic parameters. The chamber light intensity was set at 1,200 µmol m^−^^2^ s^−^^1^, with an ambient CO_2_ concentration of about 400 µmol mol^−^^1^, and relative humidity maintained between 50% and 60%. The photosynthetic parameters, Net photosynthetic rate (*Pn*), transpiration rate (*Tr*), stomatal conductance (*Gs*), and intercellular CO_2_ concentration (*C*_i_), were recorded.

The measured values were computed and interpreted based on the following definitions and equations:

**Net photosynthetic rate** (*Pn*, µmol CO_2_⋅ m^−^^2^ s^−^^1^)*,* represents the net assimilation of CO_2_ by the leaf per unit area per unit time.

\begin{eqnarray*}Pn=A=({C}_{a}\square {C}_{i})\times gc \end{eqnarray*}

where:

*C*_*a*_ = Ambient CO_2_ concentration in the leaf chamber (µmol mol^−^^1^);

*C*_*i*_ = Intercellular CO_2_ concentration (µmol mol^−^^1^);

*g*_c_ = CO_2_ conductance (mol m^−^^2^ s^−^^1^). (Note: In practice, the instrument usually directly calculates *A* (*i.e., Pn*) based on the difference in CO_2_ concentration and airflow rate.)

**Transpiration rate** (*Tr*, mmol H_2_O⋅ m^−^^2^ s^−^^1^), reflects the amount of water vapor lost through stomata per unit area per unit time.

\begin{eqnarray*}Tr={g}_{w}\times ({W}_{i}\square {W}_{a}) \end{eqnarray*}

where:

*g*_w_ = Water vapor conductance (mol H_2_O⋅ m^−^^2^ s^−^^1^);

*W*_*i*_ = Water vapor concentration inside the leaf chamber (mmol mol^−^^1^);

*W*_*a*_ = Ambient water vapor concentration (mmol mol^−^^1^).

**Stomatal conductance** (*Gs*, mol H_2_O⋅ m^−^^2^ s^−^^1^), indicates the permeability of stomata to water vapor diffusion per unit area per unit time.

\begin{eqnarray*}Gs= \frac{Tr}{VPD} \end{eqnarray*}

where:

*Tr* = Transpiration rate (mmol H_2_O⋅ m^−^^2^ s^−^^1^);

*VPD* = Vapor pressure deficit between the leaf and chamber air (kPa).

**Intercellular CO_2_ concentration** (*C*_i_, µmol mol^−^^1^), reflects the concentration of CO_2_ in the intercellular spaces of the mesophyll, indicating the CO_2_ assimilation capacity.

\begin{eqnarray*}{C}_{i}={C}_{a}\square ( \frac{Pn}{{g}_{s}/1.6} ) \end{eqnarray*}

where:

*C*_*a*_ = Ambient CO_2_ concentration (µmol mol^−^^1^)

*Pn* = Net photosynthetic rate (µmol CO_2_⋅ m^−^^2^ s^−^^1^)

*g*_s_ = Stomatal conductance (mol H_2_O⋅ m^−^^2^ s^−^^1^) (Note: Division by 1.6 accounts for the fact that stomatal conductance to water vapor is about 1.6 times of that to CO_2_.)

Contents of sodium (Na^+^, mg g^−^^1^ dry weight (DW)), potassium (K^+^, mg g^−^^1^ DW), and calcium (Ca^2^^+^, mg g^−^^1^ DW) in plant tissues were determined after dry ashing and dissolution in 1% HCl using a Shimadzu AA-7000 atomic absorption spectrophotometer (Kyoto, Japan) (Isaac & Johnson, 1985). Total nitrogen (N, mg g^−^^1^ DW) was determined using the Kjeldahl digestion method, and total phosphorus (P, mg g^−^^1^ DW) was determined using the molybdenum blue colorimetric method with absorbance measured at 880 nm (Bremner, 1965).

Proline content (µg g^−^^1^ fresh weight (FW)) was measured using the acid ninhydrin colorimetric method, with absorbance recorded at 520 nm (Bates, Waldren & Teare, 1973). Soluble sugars (mg g^−^^1^ FW), glucose (µg g^−^^1^ FW), fructose (mg g^−^^1^ FW), and sucrose (mg g^−^^1^ FW) were quantified using the anthrone method. Soluble sugars and fructose were measured at 620 nm; glucose was quantified using a glucose oxidase enzymatic reaction, and sucrose was hydrolyzed into monosaccharides before colorimetric measurement (Yemm & Willis, 1954). Soluble protein (mg g^−^^1^ FW) was measured using the Coomassie Brilliant Blue G-250 binding assay at 595 nm (Bradford, 1976).

For reactive oxygen species (ROS)-related indicators, superoxide anion (O_2_^−^, µmol g^−^^1^ FW) was measured using the nitro blue tetrazolium (NBT) reduction method, while hydrogen peroxide (H_2_O_2_, µmol g^−^^1^ FW) was quantified using an iodometric assay with absorbance at 390 nm. Electrolyte leakage was calculated from the electrical conductivity before and after leaf tissue incubation using a conductivity meter. Malondialdehyde (MDA, nmol g^−^^1^ FW) content was determined using the Thio barbituric acid (TBA) method, and absorbance was read at 532 nm and 600 nm to calculate the differential values (Heath & Packer, 1968).

The activities of the antioxidant enzymes were measured according to established methods. The specific assays for each enzyme were: superoxide dismutase (SOD, U g^−^^1^ FW) activity was measured based on the inhibition of NBT photoreduction; peroxidase (POD, U g^−^^1^ FW) activity was determined using the guaiacol-H_2_O_2_ reaction; catalase (CAT, U g^−^^1^ FW) activity was determined using the decomposition rate of H_2_O_2_, measured as the decline in absorbance; and ascorbate peroxidase (APX, U g^−^^1^ FW) activity was measured based on the oxidation of ascorbic acid, with continuous measurement at 290 nm to track the decline in absorbance over time (Aebi, 1984).

### Data processing

Data is analyzed using SPSS 22.0 software (IBM Corp., Armonk, NY, USA). Significance among treatments was tested using Duncan’s Multiple Range Test (DMRT).

## Results

### Effect of water-fertilizer management on soil electrical conductivity

Fo convenient individual reading and to avoid ambiguity, a brief review of abbreviations used for irrigation and fertilization treatment is provided as: I = conventional irrigation, T = trickle irrigation, P = film mulched trickle irrigation; F = conventional fertilizer, C = controlled-release fertilizer, and M = mixed fertilizer with F and C. Based on these definitions, the following section presents the effect of water-fertilizer management on soil electrical conductivity (EC). As shown in Table 2, different irrigation methods (I, T, P) have a significant impact on upper- and lower-layer soil electrical conductivity (EC), while fertilization methods (F, M, C) have no significant effect. During the seedling and rosette stages of Pakchoi, the upper layer soil EC of T (FT, MT, CT) and P (FP, MP, CP) treatments was significantly lower than that of the I (FI, MI, CI) treatment, and the low layer soil EC of T and P treatments was higher than that of I treatment. The upper- and lower-layer soil EC of CK control treatment is significantly lower than the other treatments. The upper layer soil EC decreased with the extension of the growth period, and there was no clear trend in the low layer soil EC.

**Table 2:** The soil EC under different irrigation and fertilization modes^a^.

Treatments	EC in upper soil layer (us⋅ cm^−1^)		EC in lower soil layer (us⋅ cm^−1^)	
	Seedling stage	Rosette stage	Seedling stage	Rosette stage
FI	1,711 ± 155 ab	1,578 ± 92 a	1,628 ± 254 c	1,690 ± 63 e
FT	1,332 ± 99 bc	1,168 ± 63 cd	2,295 ± 286 ab	2,842 ± 124 ab
FP	1,364 ± 149 bc	1,125 ± 85 d	2,385 ± 359 ab	2,285 ± 296 cd
MI	1,721 ± 202 a	1,556 ± 98 a	2,044 ± 185 bc	1,927 ± 228 de
MT	1,297 ± 162 bc	1,182 ± 87 bcd	2,599 ± 261 a	3,165 ± 385 a
MP	1,322 ± 70 bc	1,265 ± 87 bc	2,487 ± 242 ab	2,454 ± 118 c
CI	1,792 ± 216 a	1,577 ± 142 a	1,907 ± 375 bc	2,071 ± 136 d
CT	1,458 ± 157 abc	1,290 ± 65 bc	2,219 ± 242 ab	2,585 ± 145 bc
CP	1,309 ± 102 bc	1,208 ± 70 bcd	2,334 ± 213 ab	2,323 ± 76 c
CK	598 ± 50 d	319 ± 61 e	850 ± 61 d	784 ± 20 f

**Notes.**

a Number in the table is mean ± standard deviation. Different letters in a column indicate significant difference among treatments at the 5% level (*P* < 0.05, DMRT).

MP (mixed fertilizer+film mulched trickle irrigation); DW (dry weight); FW (fresh weight); FI (conventional fertilizer+conventional irrigation); FT (conventional fertilizer+trickle irrigation); FP (conventional fertilizer+film mulched trickle irrigation); MI (mixed fertilizer+conventional irrigation); MT (mixed fertilizer+trickle irrigation); CI (controlled-release fertilizer+conventional irrigation); CT (controlled-release fertilizer+trickle irrigation); CP (controlled-release fertilizer+film mulched trickle irrigation); CK (control treatment).

### Effects of Water-Fertilizer management on leaf water potential in Pakchoi

**Figure 1:**
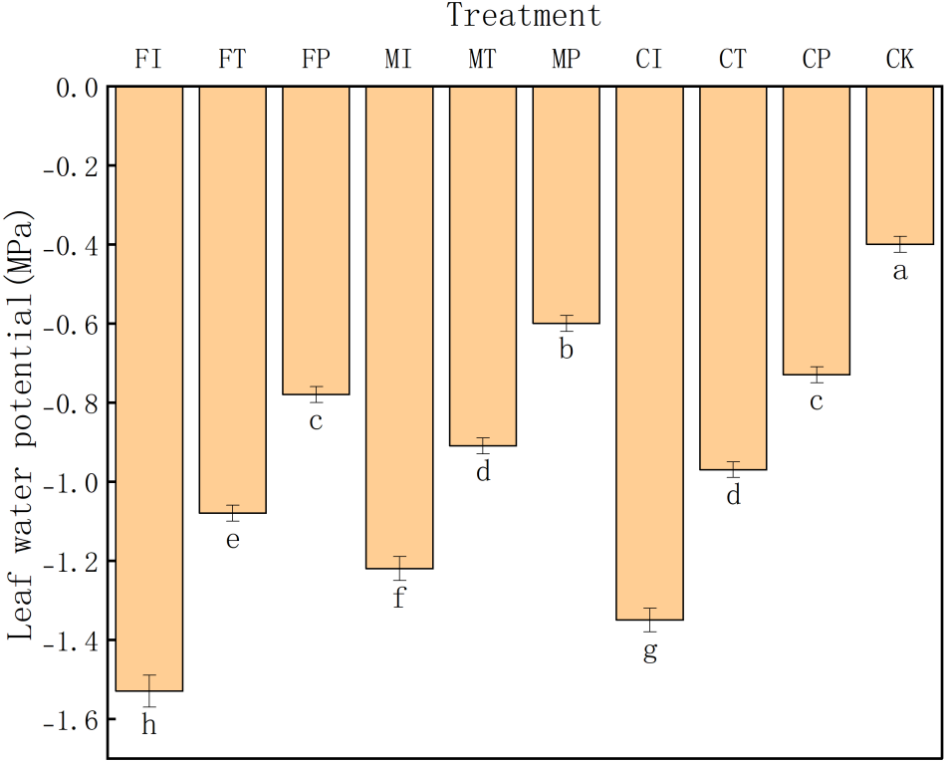
The leaf water potential of pakchoi under different irrigation and fertilization modes. Note: Lowercase letters indicate significant differences in leaf water potential among treatments at the 5% level (*P* < 0.05, DMRT).

As shown in Fig. 1, CK treatment had the highest leaf water potential (−0.40 MPa), significantly greater than that in all other treatments (significance level: a). This indicates that under salt free stress, pakchoi maintains its optimal water status without any water potential decline or related physiological stress. In contrast, FI treatment had the lowest leaf water potential (−1.53 MPa), indicating that the combination of traditional irrigation with conventional fertilizer is ineffective in maintaining plant water status under saline conditions, leading to severe water stress in the plants.

Overall, the leaf water potential values in these treatments rank as: FI < CI < MI < FT < CT < MT < FP < CP < MP < CK. This gradient distribution emphasizes the substantial impact of water-fertilizer management strategies on the regulation of leaf water potential in Pakchoi. Notably, treatments MP (−0.60 MPa) and CP (−0.73 MPa) maintained relatively high-water potential values, indicating that the combination of plastic mulching drip irrigation with mixed or controlled-release fertilizers can effectively maintain plant water and alleviate salt-induced water deficits. In contrast, low water potentials in CI (−1.35 MPa), MI (−1.22 MPa), and FT (−1.08 MPa) indicate that pakchoi plants struggle to maintain adequate hydration levels under traditional irrigation or the use of a single fertilizer type.

Although controlled-release fertilizers (CI, CT, CP) are designed to gradually release nutrients and stabilize plant water status, their effectiveness under saline conditions varied depending on the irrigation method. For example, CI treatment had a relatively low water potential (−1.35 MPa), and CP treatment achieved a significantly higher value (−0.73 MPa). This indicates that the regulation of controlled-release fertilizers depends on compatible and efficient water delivery regimes.

### Effects of water-fertilizer management on the contents and ratios of Na^+^, K^+^, and Ca^2+^ in pakchoi

This experiment investigated the effects of different water-fertilizer management strategies on K^+^, Na^+^, and Ca^2+^ contents in pakchoi, as well as the ratios of K^+^/Na^+^ and Na^+^/Ca^2+^. As shown in Table 3, there are significant differences in these indicators of treatments, highlighting the significant impact of water-fertilizer management on ion absorption and balance in Pakchoi.

**Table 3:** The Na^+^, K^+^, Ca^2+^ contents of single plant and their ratio under different irrigation and fertilization modes^a^.

Treatments	K^+^(%)	Ca^2+^ (%)	Na^+^(%)	K^+^/Na^+^	Na^+^/Ca^2+^
FI	1.32 ± 0.14 g	0.41 ± 0.03 g	9.22 ± 0.29 a	0.14 ± 0.01 g	22.64 ± 2.13 a
FT	2.36 ± 0.28 de	0.67 ± 0.02 e	7.08 ± 0.43 b	0.33 ± 0.05 e	10.53 ± 0.59 cd
FP	1.78 ± 0.14 f	0.65 ± 0.02 e	4.9 ± 0.15 d	0.36 ± 0.03 e	7.6 ± 0.22 e
MI	2.13 ± 0.28 e	0.38 ± 0.02 g	6.62 ± 0.42 bc	0.32 ± 0.04 e	17.28 ± 1.70 b
MT	4.06 ± 0.43 a	0.83 ± 0.02 c	5.07 ± 0.35 d	0.81 ± 0.09 b	6.15 ± 0.47 f
MP	3.28 ± 0.43 bc	0.91 ± 0.02 b	3.5 ± 0.56 e	0.96 ± 0.16 b	3.84 ± 0.61 g
CI	1.39 ± 0.15 g	0.56 ± 0.02 f	6.06 ± 0.41 c	0.23 ± 0.04 f	10.82 ± 0.90 cd
CT	2.83 ± 0.29 cd	0.71 ± 0.02 d	6.67 ± 0.56 bc	0.42 ± 0.06 d	9.43 ± 0.84 d
CP	3.73 ± 0.86 ab	0.83 ± 0.02 c	6.39 ± 0.41 bc	0.59 ± 0.12 c	7.72 ± 0.70 e
CK	3.89 ± 0.42 ab	0.98 ± 0.03 a	1.12 ± 0.28 f	3.61 ± 0.86 a	1.15 ± 0.28 h

**Notes.**

a Number in the table is mean±standard deviation.Different letters in a column indicate significant difference among treatments at the 5% level (*P* < 0.05, DMRT).

MP (mixed fertilizer+film mulched trickle irrigation); DW (dry weight); FW (fresh weight); FI (conventional fertilizer+conventional irrigation); FT (conventional fertilizer+trickle irrigation); FP (conventional fertilizer+film mulched trickle irrigation); MI (mixed fertilizer+conventional irrigation); MT (mixed fertilizer+trickle irrigation); CI (controlled-release fertilizer+conventional irrigation); CT (controlled-release fertilizer+trickle irrigation); CP (controlled-release fertilizer+film mulched trickle irrigation); CK (control treatment).

First, there are significant differences in K^+^ content in these treatments. K^+^ content in CK (3.89%), MP (3.28%), MT (4.06%), and CP (3.73%) treatments was higher than other treatments. These treatments accumulated significantly more K^+^ than FI (1.32%) and FT (2.36%), indicating that optimized water-fertilizer management can effectively promote K^+^ absorption and accumulation, enhancing water-salt balance ability of plants. FI treatment had the lowest K^+^ content, reflecting the inhibitory effect of traditional water-fertilizer management on K^+^ absorption.

The trend of Na^+^ content was similar to that of K^+^. Na^+^ content in MP (3.5%) and CP (6.39%) was significantly lower than that in FI treatment (9.22%). This indicates that optimized water-fertilizer management strategies can effectively mitigate salt stress, reduce Na^+^ accumulation, and improve the ion balance in pakchoi. In contrast, high Na^+^ content in FI treatment indicates excessive Na^+^ accumulation, which may lead to increased salt stress in plants.

In terms of Ca^2+^ content, CK treatment had the highest Ca^2+^ content (0.98%), significantly higher than the other treatments. MP and CP treatments also had relatively high Ca^2+^ content (0.91% and 0.83%, respectively), indicating that these optimized water-fertilizer management practices can effectively promote Ca^2+^ absorption and accumulation. This is crucial for enhancing plant cell wall stability and stress resistance. On the other hand, FI and MI treatments had low Ca^2+^ content, indicating that these management strategies may lead to insufficient calcium absorption.

For ion ratios, the K^+^/Na^+^ ratio was the highest in MP treatment (0.94), and the lowest in FI treatment (0.14). Treatments such as MP and CP can effectively increase the utilization of K^+^ and reduce Na^+^ accumulation, thereby enhancing the resistance to salt stress in plants. Low K^+^/Na^+^ ratio in FI treatment indicates a strong salt stress response in Pakchoi and poor ion balance under traditional irrigation and fertilization methods.

The Na^+^/Ca^2+^ ratio was the highest in FI treatment (22.55), indicating that traditional water-fertilizer management may lead to excessive Na^+^ accumulation and disrupt the balance of calcium under salt stress. The Na^+^/Ca^2+^ ratio was significantly low in MP and CP treatments, indicating that these treatments can effectively improve the negative impact of salt stress on the ion balance of plants.

Optimized water-fertilizer management strategies such as the MP and CP treatments can significantly increase K^+^ accumulation, reduce Na^+^ accumulation, and improve Ca^2+^ absorption to maintain ion balance in plants and effectively alleviate salt stress, thereby improving salt tolerance of pakchoi. Traditional water-fertilizer management strategies such as FI treatment may lead to high Na^+^ accumulation and low K^+^ content, increasing the risk of salt stress.

### Effects of water-fertilizer management on nitrogen and phosphorus content in pakchoi plants

**Figure 2:**
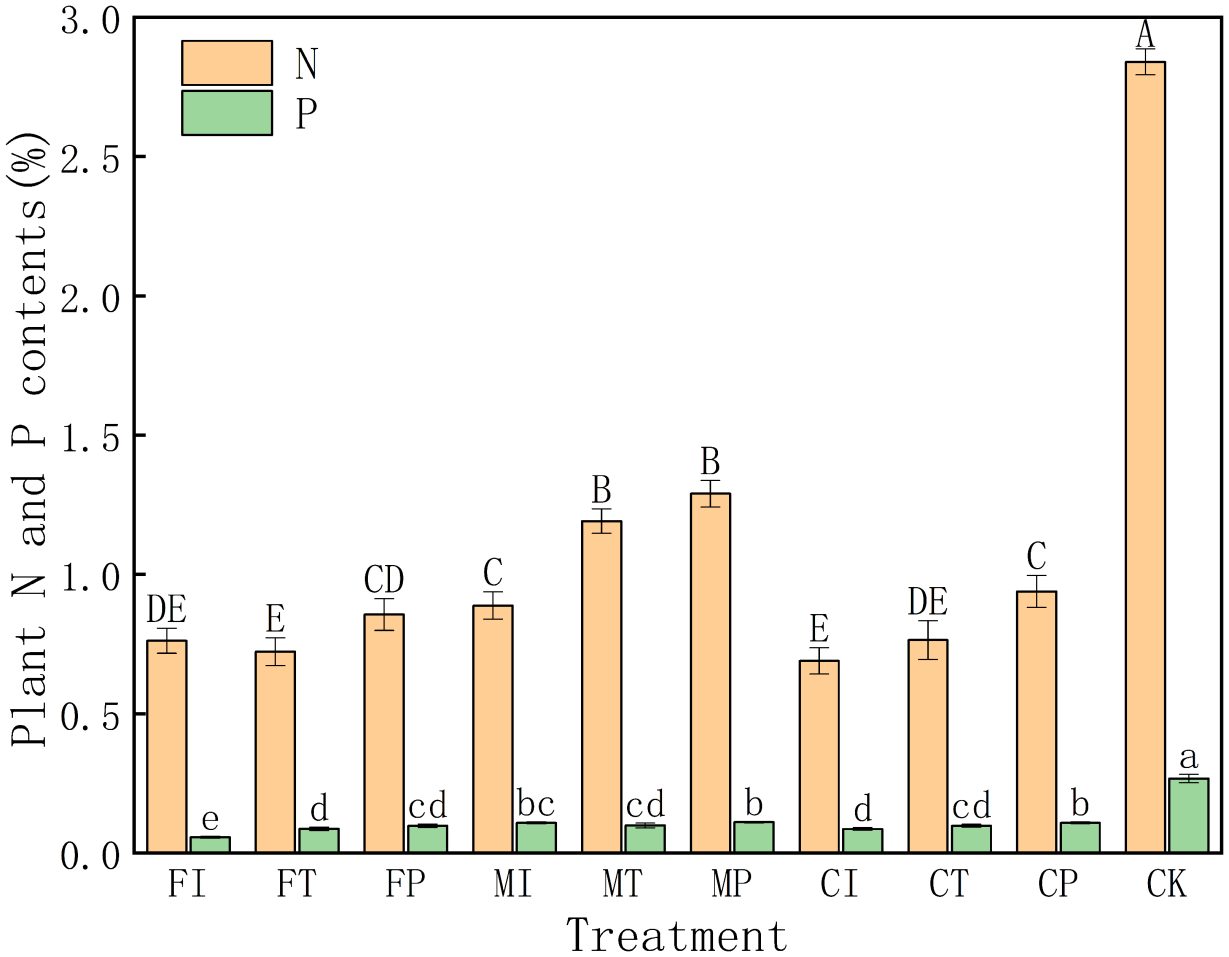
Effects of different irrigation and fertilization modes on nitrogen and phosphorus content in pakchoi plants. Note: Capital letters above the bars indicate significant differences in nitrogen content among treatments at the 5% level. Lowercase letters indicate significant differences in phosphorus content among treatments at the 5% level (*P* < 0.05, DMRT).

As shown in Fig. 2, the nitrogen (N) content in CK treatment is significantly higher than all other treatments (2.84%), indicating that salt stress can substantially inhibit nitrogen uptake in pakchoi. N content was generally low under salt stress, particularly in FI (0.762%), FT (0.723%), and CI (0.690%) treatments, reflecting severe suppression of nitrogen accumulation in these groups. In contrast, these treatments such as MP (1.29%), MT (1.19%), and MI (0.89%) had relatively high N levels, indicating that the combination of plastic mulching drip irrigation and mixed fertilizer can effectively mitigate salt damage and enhance nitrogen uptake.

There is a similar trend for phosphorus (P) content. The CK group has a higher P content (0.268%) than all other treatments. The P level was the lowest in FI treatment (0.0567%), and P levels of traditional irrigation (FI) and controlled-release fertilizer (CI) treatments were low at 0.0567% and 0.0866%, respectively. Conversely, P contents in MP (0.111%), CP (0.109%), and MI (0.109%) treatments were relatively high. This indicates that the combination of plastic mulching drip irrigation with mixed or controlled-release fertilizers can significantly improve phosphorus uptake.

The data indicates that salt stress can significantly inhibit nitrogen and phosphorus absorption in pakchoi, especially under traditional irrigation and single fertilizer. In all treatments, the combination of plastic mulching drip irrigation and mixed fertilizer (MP) has the most pronounced effect in alleviating salt stress and enhancing nutrient accumulation.

### Effects of water-fertilizer management on organic solute accumulation in pakchoi plants

**Figure 3:**
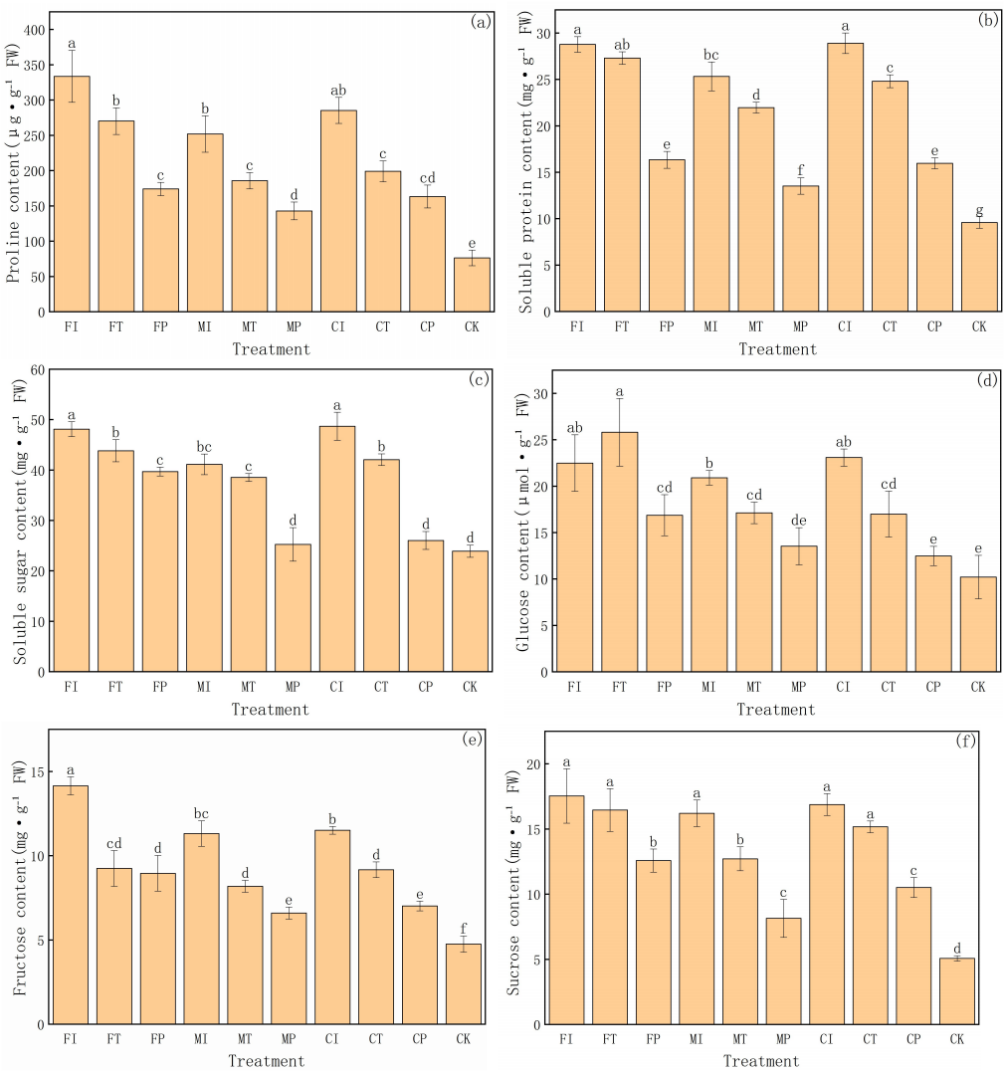
The organic solute accumulation in Pakchoi under different irrigation and fertilization modes: (A) Proline content, (B) Soluble protein content, (C) Soluble sugar content, (D) Glucose content, (E) Fructose content, (F) Sucrose content. Note: Lowercase letters indicate significant differences in organic solute accumulation l among treatments at the 5% level (*P* < 0.05, DMRT).

This study further evaluates the accumulation of various organic solutes in pakchoi under different water-fertilizer management systems, with a focus on six physiological indicators: proline, soluble protein, soluble sugar, glucose, fructose, and sucrose (Fig. 3). These solutes are critical for osmoregulation and stress adaptation under saline conditions. The significant differences between different treatments and the distribution of values in groups highlight the substantial impact of water-fertilizer strategies on the osmotic adjustment capacity and metabolic activity of pakchoi.

Proline content (Fig. 3A) was the highest in FI treatment (333.68 µg g^−1^ FW), and the CK group was the lowest (76.43 µg g^−1^ FW). This indicates that salt stress can significantly induce proline biosynthesis in Pakchoi, facilitating osmotic regulation and protecting cells from dehydration. It is worth noting that these treatments such as MP and CP had lower proline levels than FI, FT, and CI. This indicates that plastic mulching drip irrigation combined with optimized fertilization can alleviate stress intensity and reduce the need for excessive accumulation of protective solutes.

In terms of soluble protein content (Fig. 3B), FI and CI treatments had the highest levels (28.78 and 28.91 mg g^−1^ FW, respectively), with values significantly greater than those recorded under MP, FP, CP, and CK treatments. Due to the contribution of soluble proteins to osmotic balance and stress response, their elevated levels in high salinity treatment reflect the intensity of cellular metabolic responses under salt stress. On the contrary, the CK group had the lowest protein content (9.59 mg g^−1^ FW), consistent with its unstressed condition and lack of induced protein expression. MP treatment also showed relatively low protein accumulation (13.53 mg g^−1^ FW), further confirming its efficacy in mitigating environmental stress.

Soluble sugars (Fig. 3C) are another key osmotic regulator and plays an essential role in stress response. FI and CI treatments had soluble sugar concentrations exceeding 48 mg g^−1^ FW, significantly higher than that of the CK group (23.91 mg g^−1^ FW). This indicates that under severe salt stress, Pakchoi accumulates sugars to maintain cellular osmotic potential. Meanwhile, MP and CP treatments decreased in sugar content (25.26 and 26.04 mg g^−1^ FW, respectively), indicating that these practices help alleviate salt-induced osmotic stress.Glucose (Fig. 3D) and fructose (Fig. 3E) levels have a similar trend. The glucose content was the highest in FT treatment (25.82 µmol g^−1^ FW), and MP and CK had the lowest levels (13.54 and 10.22 µmol g^−1^ FW, respectively). Elevated glucose levels in FI, CI, and MI indicate that salt stress can activate carbohydrate metabolism pathways, leading to enhanced glucose synthesis. Similarly, fructose concentrations were significantly higher in FI and CI (14.15 and 11.51 mg g^−1^ FW, respectively), with CK showing the lowest accumulation (4.76 mg g^−1^ FW) again. These results reflect the proline pattern and highlight the role of sugar metabolism in maintaining cellular water balance under saline conditions.

Sucrose content (Fig. 3F) further substantiates the above findings. The levels of treatments FI, CI, FT, MI, and CT were significantly high (16–17.5 mg g^−1^ FW), while the levels of MP and CK were the lowest (8.15 and 5.08 mg g^−1^ FW, respectively). These differences indicate that plants have a low demand for sucrose-mediated osmotic adjustment under lower or no stress conditions.

FI and CI treatments consistently had the highest concentrations of osmotic regulators, indicating that traditional irrigation combined with conventional or controlled-release fertilizers resulted in severe salt stress in pakchoi. In contrast, the accumulation of MP and CP treatments on multiple parameters was low, indicating that the combination of plastic mulching drip irrigation and optimized fertilization can effectively reduce stress intensity and minimize plant dependence on osmoregulatory solutes. The CK group had the lowest values in all indicators, further validated that salt stress is the main driving factor for organic solute accumulation in pakchoi.

### Effects of water-fertilizer management on ROS, MDA content, and electrolyte leakage in pakchoi plants

**Figure 4:**
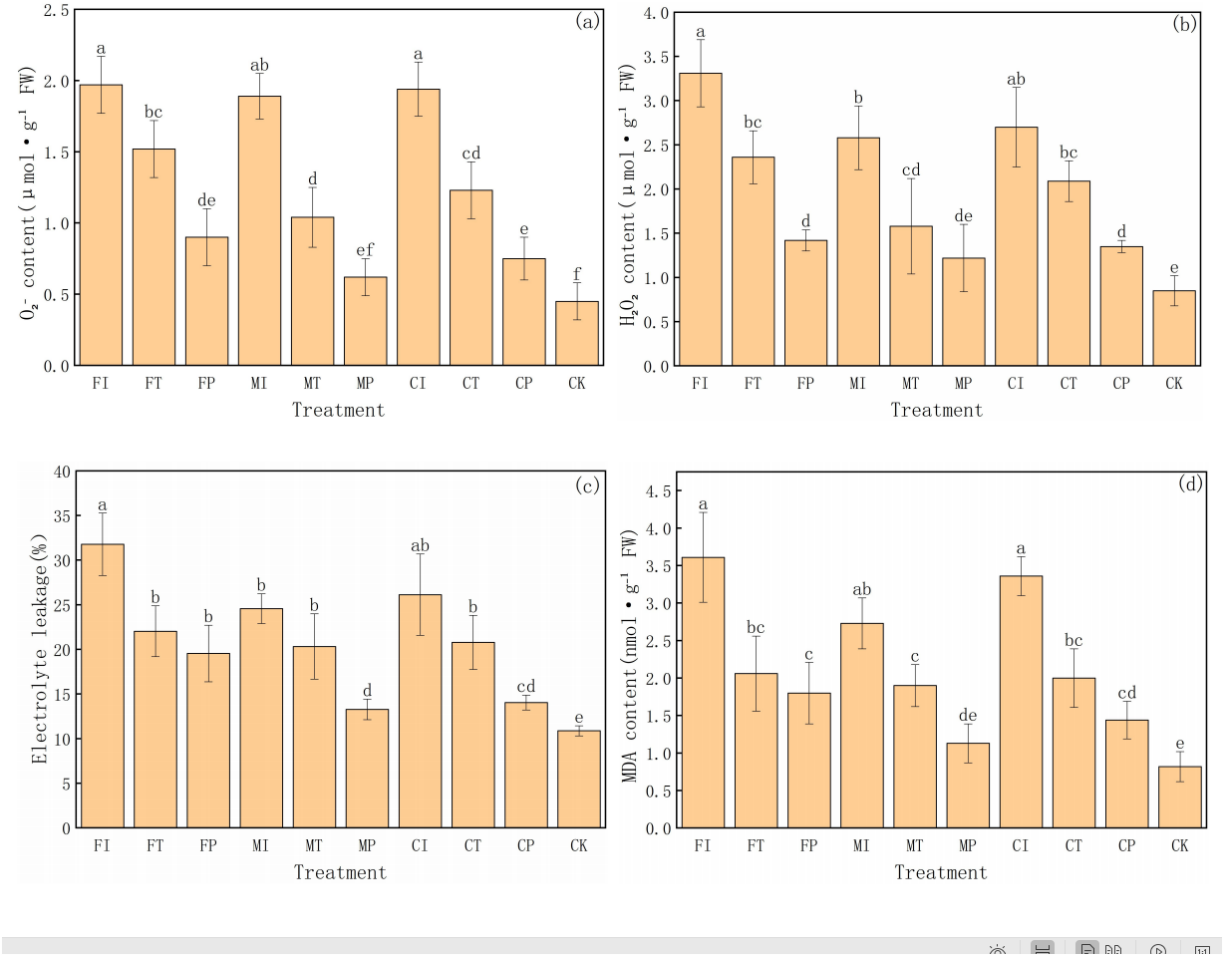
The ROS, MDA contents, and electrolyte leakage in Pakchoi under different irrigation and fertilization modes: (A) ${\mathrm{O}}_{2}^{-}$ content, (B) H_2_O_2_ content, (C) Electrolyte leakage, (D) MDA content. Note: Lowercase letters indicate significant differences in ROS, MDA content, and electrolyte leakage among treatments at the 5% level (*P* < 0.05, DMRT).

This experiment further analyzed the degree of oxidative damage and membrane stability in pakchoi under salt stress by measuring four key physiological indicators: superoxide anion (O_2_^−^), hydrogen peroxide (H_2_O_2_) content, electrolyte leakage, and malondialdehyde (MDA) content (Fig. 4). These indicators reflect the accumulation of reactive oxygen species (ROS) and lipid peroxidation of membranes under stress. The results reveal significant differences in treatments, highlighting the substantial impact of water-fertilizer management strategies on alleviating salt stress and enhancing cellular protection mechanisms.

First, O_2_^−^ content was the highest in FI treatment (1.97 µmol g^−1^ FW), significantly higher than in FP, MP, CP, and CK treatments, and the CK group was the lowest (0.45 µmol g^−1^ FW). This indicates a gradient shift from high salt stress to no stress, as shown in Fig. 4A. Similarly, O_2_^−^ content in CI, MI, and FT treatments was relatively high, indicating significant accumulation of ROS under traditional irrigation or single fertilizer systems. In contrast, MP and CP treatments decreased in O_2_^−^ content, indicating that the combination of plastic mulching drip irrigation with optimized fertilization can effectively inhibit ROS generation and reduce oxidative damage.

The trend in H_2_O_2_ content further supports these findings (Fig. 4B). FI treatment had the highest H_2_O_2_ level (3.31 µmol g^−1^ FW), followed by CI (2.70 µmol g^−1^ FW) and MI (2.58 µmol g^−1^ FW), with MP (1.22 µmol g^−1^ FW) and CK (0.85 µmol g^−1^ FW) being the lowest. The significant inhibition of H_2_O_2_ accumulation in MP and CP treatments highlights their important role in mitigating salt-induced ROS accumulation.

Electrolyte leakage is an important indicator of cell membrane integrity and follows the same trend as the oxidative stress markers (Fig. 4C). The electrolyte leakage rate in FI treatment was the highest (31.77%), and the CK group was the lowest (10.88%), indicating enhanced membrane damage and leakage under salt stress. MP treatment had a leakage rate of 13.28%, slightly higher than CK and significantly lower than in high-stress treatments (FI, CI, FT), indicating the protective effect of this treatment on cell membrane. CP treatment had a relatively low leakage rate (14.05%), further indicating that optimized water-fertilizer management can significantly reduce membrane damage under salt stress.

MDA content is a key indicator of lipid peroxidation and has similar trends (Fig. 4D). MDA content was the highest in FI treatment (3.61 nmol g^−1^ FW), and the lowest in CK treatment (0.82 nmol g^−1^ FW). CI treatment had high levels of MDA (3.36 nmol g^−1^ FW), indicating severe membrane lipid peroxidation under these conditions. In contrast, MDA content in MP and CP treatments was significantly low at 1.13 and 1.44 nmol g^−1^ FW, respectively, further validating the effectiveness of optimized water-fertilizer management strategies in reducing membrane oxidative damage and enhancing stress tolerance of pakchoi.

FI and CI treatments had significantly high values in all oxidative stress-related indicators, reflecting that the combination of traditional irrigation with conventional or controlled-release fertilizers cannot effectively protect cells under salt stress, leading to increased oxidative damage. In contrast, MP and CP treatments had low values in all indicators, indicating their good ability to reduce ROS accumulation and mitigate membrane system damage, making them the most effective management strategies for alleviating salt stress. The CK group consistently showed the lowest values in all indicators, further confirming that that salt stress is the main driving factor for oxidative damage and cell membrane damage.

### Effects of water-fertilizer management on antioxidant enzyme activities in pakchoi

**Figure 5:**
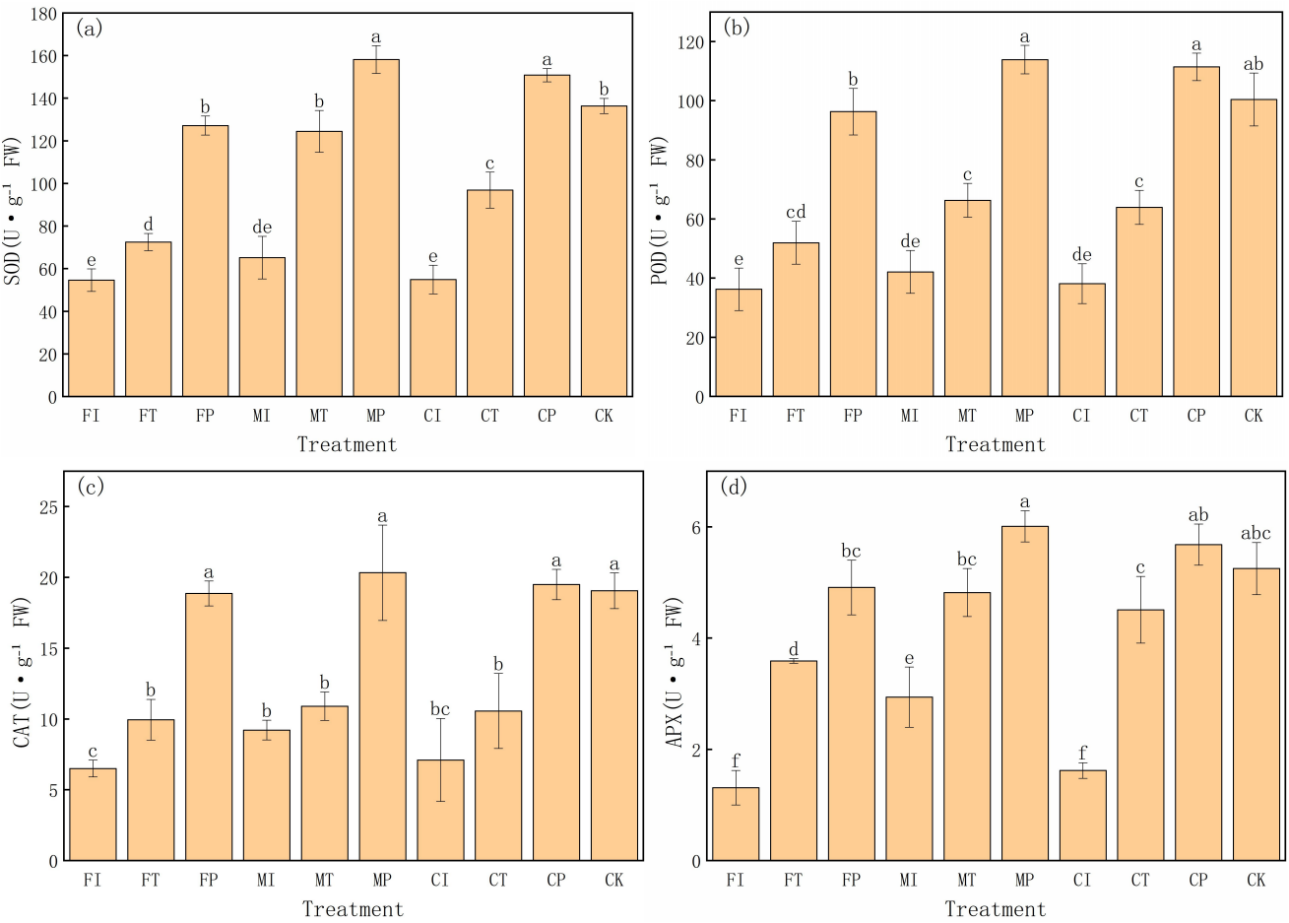
The antioxidant enzyme activity in Pakchoi under different water-fertilizer management modes: (A) SOD activity, (B) POD activity, (C) CAT activity, (D) APX activity. Note: Lowercase letters indicate significant differences in antioxidant enzyme activities among treatments at the 5% level (*P* < 0.05, DMRT).

This experiment also evaluated the effects of different water-fertilizer management strategies on the antioxidant enzyme activities in Pakchoi. The activities of superoxide dismutase (SOD), peroxidase (POD), catalase (CAT), and ascorbate peroxidase (APX) (Fig. 5) are specifically analyzed. The results showed significant differences in enzyme activities in different treatments, highlighting the impact of water-fertilizer management on the oxidative stress response of pakchoi. SOD activity is a key enzyme in the elimination of superoxide anion radicals. It was the highest in MP treatment (158.19 U g^−1^ FW), followed by CP (150.85 U g^−1^ FW), both significantly higher than the other treatments. The SOD activity in FI treatment was the highest (54.66 U g^−1^ FW), significantly lower than that of MP and CP treatments. This trend suggests that under optimized water-fertilizer management conditions (MP and CP treatments), the oxidative stress response of pakchoi is significantly enhanced, and the response under traditional management practices (FI treatment) is weak (Fig. 5A).

Similarly, POD activity was the highest in MP treatment (113.91 U g^−1^ FW), followed by CP (111.46 U g^−1^ FW), both significantly higher than the other treatments. The POD activity was the lowest in FI treatment (36.28 U g^−1^ FW), which aligns with its lower oxidative stress response (Fig. 5B).

CAT activity was the highest in MP treatment (20.33 U g^−1^ FW), followed by CP treatment (19.50 U g^−1^ FW), and CAT activity was the lowest in FI treatment (6.51 U g^−1^ FW). This indicates that optimized water-fertilizer management (such as MP and CP treatments) can significantly improve the resistance of Pakchoi to detoxify hydrogen peroxide (H_2_O_2_), thereby providing better antioxidant damage protection (Fig. 5C).

APX activity, as an enzyme responsible for reducing H_2_O_2_ to water, was the highest in MP treatment (6.01 U g^−1^ FW), followed by CP (5.68 U g^−1^ FW). FI treatment had the lowest APX activity (1.31 U g^−1^ FW), indicating that under traditional management practices, the activity of ascorbate peroxidase is low, leading to weak H_2_O_2_ detoxification capacity (Fig. 5D).

These results indicates that water-fertilizer management strategies, particularly MP and CP treatments, can significantly enhance the antioxidant defense system in Pakchoi, improving its resistance to oxidative damage under salt stress. Traditional treatments (FI and CI) consistently showed low enzyme activities, indicating a weak response to oxidative stress.

### Effects of water-fertilizer management on chlorophyll content in pakchoi

**Figure 6:**
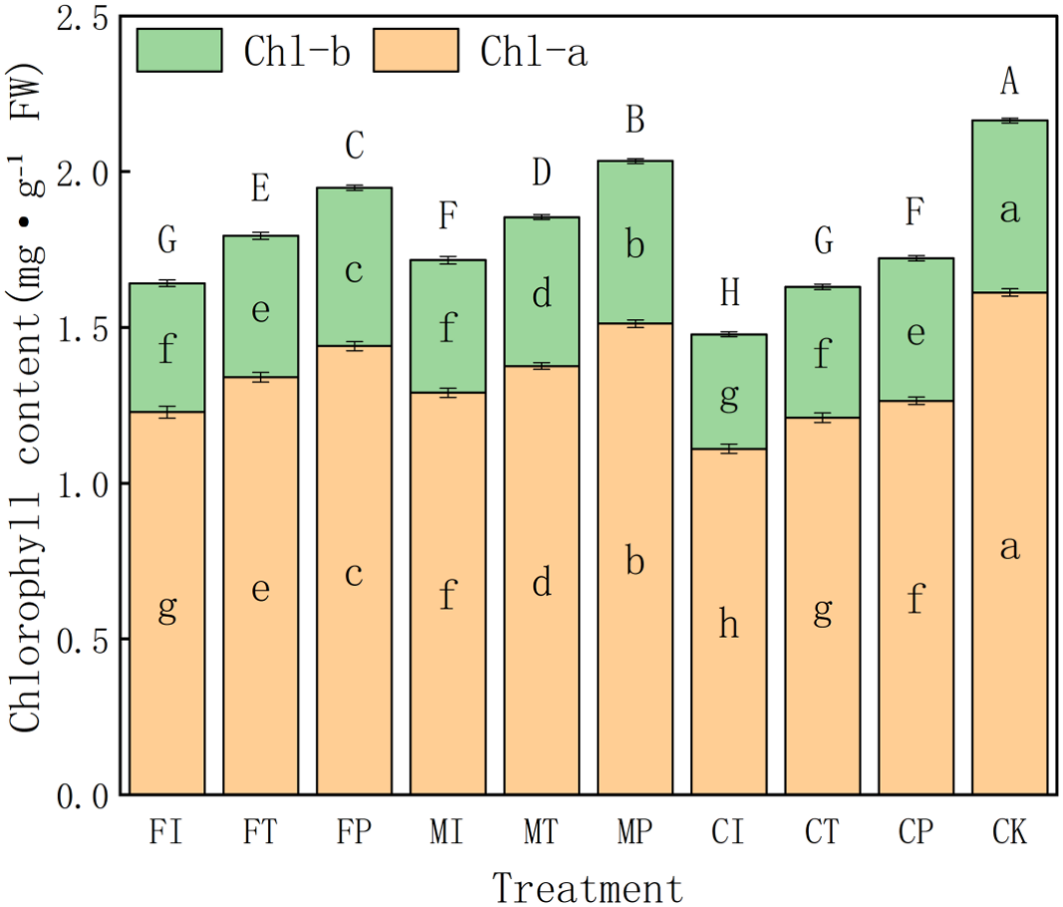
The chlorophyll content of pakchoi under different water-fertilizer management regimes. Note: Different capital letters above the bars indicate significant differences in total chlorophyll content among treatments at the 5% level. Different lowercase letters indicate significant differences in chlorophyll a or b content among treatments at the 5% level (*P* < 0.05, DMRT).

The effects of different water-fertilizer management practices on the chlorophyll a (Chl-a), chlorophyll b (Chl-b), and total chlorophyll (Chl-a + Chl-b) contents in pakchoi leaves are further analyzed. The results showed significant differences in treatments of all three indicators, indicating that water-fertilizer management plays a critical role in regulating the photosynthetic potential and physiological activity of Pakchoi (Fig. 6).

In terms of Chl-a content, CK treatment had the highest value (1.612), followed by MP (1.512) and FP (1.44), indicating that salt free stress and optimized water-fertilizer management (such as MP treatment) can promote the synthesis of photosynthetic pigments. In contrast, CI (1.11) and FI (1.228) treatments had the lowest Chl-a contents, indicating that the combination of traditional irrigation with conventional fertilizers may inhibit chlorophyll synthesis, which may be due tosalt stress and suboptimal nutrient availability.

The trend of changes in Chl-b content is consistent with the trend of changes in Chl-a. The CK group had the highest value (0.552), followed by MP (0.522) and FP (0.508), all significantly higher than FI (0.414) and CI (0.368). This further confirms that optimized water-fertilizer management can effectively enhance chlorophyll accumulation. Intermediate Chl-b values in CT and MI treatments indicates that partially improved management measures (such as controlled-release fertilizers) provide moderate stress alleviation benefits.

For total chlorophyll content (Chl-a + Chl-b), CK treatment had the highest level (2.16), followed by MP (2.03), both significantly higher than all other treatments. This indicates that optimized water-fertilizer strategies or non-saline environments can significantly improve photosynthetic capacity in pakchoi. In contrast, CI had the lowest total chlorophyll content (1.48), followed by FI (1.64) and CT (1.63). This indicates that traditional management approaches can impair chlorophyll biosynthesis, which may hinder plant growth and yield formation.

The overall trends highlight the effectiveness of optimized water-fertilizer regimes, especially the MP treatment (the combination of plastic mulching drip irrigation with mixed fertilizers), which significantly increased the chlorophyll content in Pakchoi. Its chlorophyll levels were only second to non-salt CK treatment and significantly better than those under traditional irrigation (FI) and single-fertilizer treatments (CI). These findings are consistent with earlier results, indicating that the MP treatment has the ability to alleviate salt stress and enhance stress resilience, further confirming the core role of water-fertilizer management in regulating the physiological metabolism of pakchoi.

### Effects of water-fertilizer management on photosynthetic parameters of pakchoi

**Figure 7:**
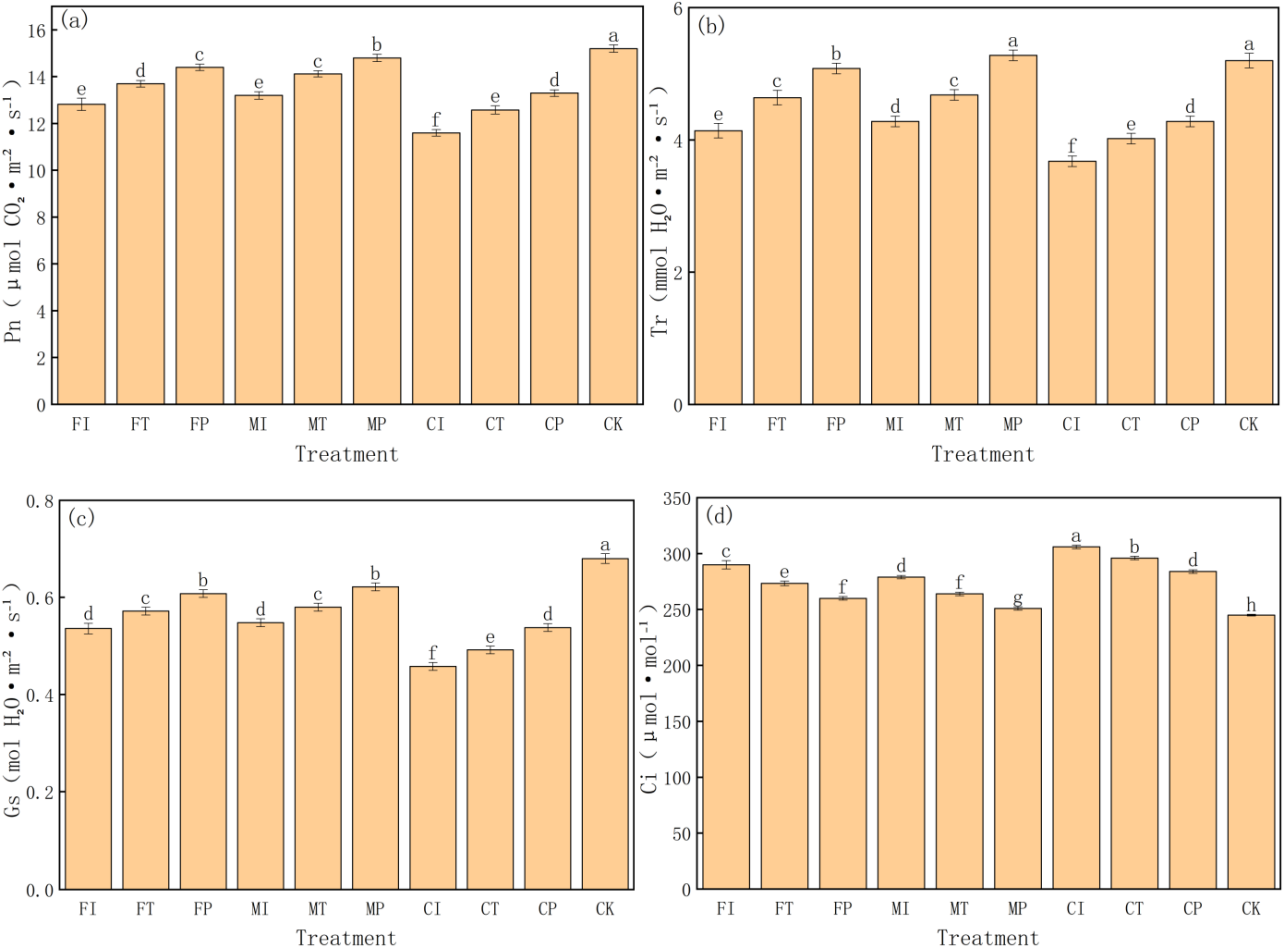
The photosynthetic parameters of pakchoi under different water-fertilizer management regimes: (A) *P*_*n*_, (B) *T*_*r*_, (C) *G*_*s*_, (D) *Ci*. Note: Different lowercase letters indicate significant differences in photosynthetic parameters among treatments at the 5% level (*P* < 0.05, DMRT).

This study systematically evaluates the regulation of different water-fertilizer management strategies on key photosynthetic parameters in pakchoi, including net photosynthetic rate (*Pn*), transpiration rate (*Tr*), stomatal conductance (*Gs*), and intercellular CO_2_ concentration (*C*_*i*_). The experimental data revealed significant differences in all four parameters of treatments, indicating that water-fertilizer management will significantly affect the gas exchange and photosynthetic efficiency of pakchoi (Fig. 7).

First, for *Pn* (Fig. 7A), CK treatment had the highest value (15.2 µmol CO_2_⋅ m^−^^2^ s^−^^1^), followed by MP (14.8) and FP (14.4), and CI treatment had the lowest value (11.6). This indicates that under non-saline conditions (CK) and optimized water-fertilizer strategies (*e.g.*, MP and FP), photosynthetic efficiency in Pakchoi is significantly enhanced. Conversely, low photosynthetic rates in CI and FI treatments indicate that the combination of traditional irrigation with conventional fertilizers under high salinity conditions will significantly inhibit photosynthesis.

*Tr* has a similar trend to that of *Pn* (Fig. 7B). MP treatment had the highest transpiration rate (5.28 mmol H_2_O⋅ m^−^^2^ s^−^^1^), which was not significantly different from CK (5.2) and significantly higher than CI (3.68) and FI (4.14). This indicates that optimized water-fertilizer management can improve water uptake and transpiration, help regulate leaf temperature and maintain photosynthetic balance. Low *Tr* may reflect stomatal closure under salt stress, limiting CO_2_ intake and inhibiting photosynthesis.

For *Gs* (Fig. 7C), CK treatment had the highest value (0.68 mol H_2_O⋅ m^−^^2^ s^−^^1^), followed by MP (0.622), and CI had the lowest (0.458). This pattern is consistent with the trends in *Pn* and *Tr*. MP and FP treatments also increased significantly in *Gs*, showing their role in promoting stomatal opening and enhancing photosynthetic capacity. In contrast, treatments such as CI and CT had low *Gs*, which limited CO_2_ exchange efficiency, indicating limited support for photosynthesis.

*C*_*i*_ was the highest in CI treatment (306 µmol mol^−^^1^), and CK had the lowest *C*_*i*_ (245) (Fig. 7D). This parameter reflects the balance between stomatal aperture and CO_2_ assimilation efficiency. High *C*_*i*_ in CI, combined with its low *Pn* and *Gs*, indicates poor carbon assimilation ability despite CO _2_ accumulation. In contrast, CK and MP treatments had the lowest *C*_*i*_ and the highest *Pn*, indicating highCO_2_ utilization efficiency and stronger photosynthetic activity.

MP treatment had the most favorable photosynthetic performance under salt stress, characterized by high *Pn*, *Tr*, and *Gs* values and low *C*_*i*_. This indicates optimal gas exchange capacity and CO_2_ assimilation efficiency, making it an effective strategy for optimizing photosynthetic function.

Although CK performed the best, it is a non-saline control, further validating the effectiveness of salt stress on photosynthesis. CI, FI, and CT treatments consistently showed low performance in all parameters, highlighting the limitations of traditional water-fertilizer management in mitigating salt-induced photosynthetic inhibition.

### Effects of water-fertilizer management on pakchoi plant height

**Figure 8:**
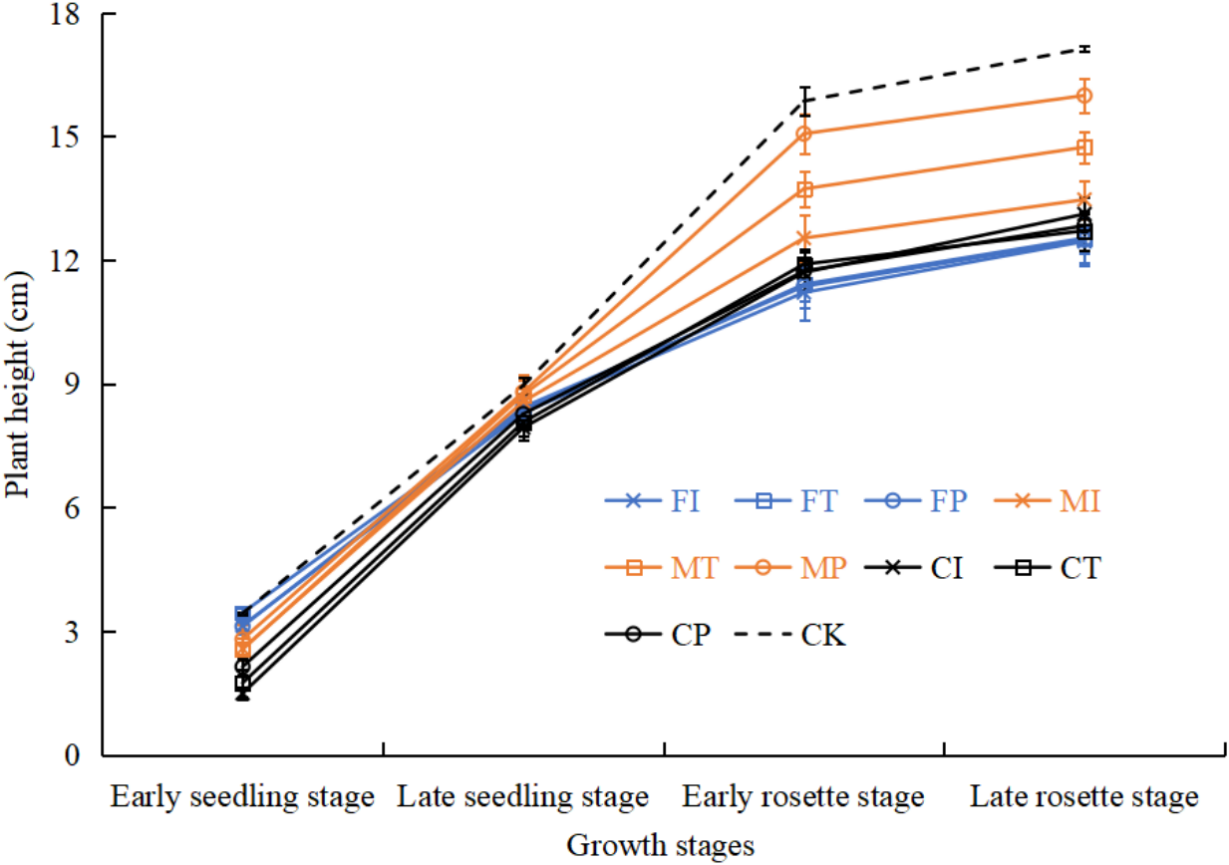
The plant height of pakchoi under different irrigation and fertilization modes.

As shown in Fig. 8, in the growth period of pakchoi extending, the plant height of pakchoi in all treatments gradually increased. Plant heights in treatments were significantly different in the later stages of pakchoi seedlings. CK control treatment had taller pakchoi plants than salt-stressed treatments. M treatment had taller pakchoi plants than C and F treatments. Under salt-stressed treatments, MP treatment had the tallest plants. MT treatment was significantly taller than MI treatment, and MI was slightly taller than C (CI, CT, CP) and F (FI, FT, FP) treatments. There were no significant differences between C and F treatments.

### Effects of water-fertilizer management on pakchoi biomass

As shown in Table 4, pakchoi had the highest fresh and dry weights under CK control treatment, significantly greater than salt-stressed treatments. In the salt-stressed treatments, MP treatment had the highest fresh weight, and its dry weight was significantly greater than that of F and C treatments. The fresh and dry weights of MP treatment were 15% and 3% higher than those of MT, 19% and 7% higher than those of MI, 21% and 12% higher than those of CP, 23% and 9% higher than those of CT, 22% and 20% higher than those of CI, 24% and 18% higher than those of FP, 26% and 20% higher than those of FT, and 39% and 42% higher than those of FI.

**Table 4:** The biomass of pakchoi under different irrigation and fertilization modes^a^.

Treatments	Fresh matter (g)	Dry matter (g)
FI	32.47 ± 1.89 e	2.09 ± 0.18 g
FT	35.86 ± 1.19 d	2.46 ± 0.14 f
FP	36.54 ± 1.45 cd	2.5 ± 0.15 ef
MI	38.12 ± 0.81 c	2.77 ± 0.11 bcd
MT	39.39 ± 1.23 c	2.88 ± 0.07 bc
MP	45.26 ± 2.04 b	2.96 ± 0.12 b
CI	37.19 ± 0.71 c	2.47 ± 0.11 f
CT	36.75 ± 1.12 cd	2.71 ± 0.07 cde
CP	37.47 ± 1.63 c	2.64 ± 0.12d ef
CK	49.38 ± 0.46 a	3.46 ± 0.06 a

**Notes.**

a Number in the table is mean ± standard deviation. Different letters in a column indicate significant difference among treatments at the 5% level (*P* < 0.05, DMRT).

## Discussion

The effects of various water-fertilizer management strategies on the growth, physiological, and biochemical responses of pakchoi under salt stress are evaluated. The results revealed that optimized treatments, especially MP (plastic film mulched drip irrigation combined with mixed fertilizer), can significantly alleviate salt-induced damage, enhance photosynthetic capacity, maintain ionic balance, reduce oxidative injury, and increase biomass yield. Based on 11 different physiological and morphological dimensions (2.1–2.11), this study provides robust empirical support for crop salt tolerance strategies.

### Phenotypic performance: significant improvements in growth, biomass, and water status

Under salt stress, MP treatment showed the most optimal growth, with plant height and fresh and dry biomass significantly exceeding all other salt-treated groups, even approaching the levels in the non-saline control (CK) (Fig. 8, Table 4). These increases were statistically significant (*p* < 0.05), with a 39% (fresh weight) and 42% (dry weight) increase in biomass compared to FI, indicating a significant effect. Chen, Zhang & Wang (2016) demonstrated that drip irrigation combined with slow-release fertilizers can significantly improve the height and yield of bell pepper under salinity stress. It is worth noting that even at moderate salinity (0.3% NaCl), our study observed these benefits, indicating strong adaptability through the application of MP.

Leaf water potential data further indicated that MP treatment can effectively mitigate salt-induced dehydration stress (−0.60 MPa), second only to the CK group (−0.40 MPa), and the lowest in FI treatment (−1.53 MPa), indicating poor water balance under conventional irrigation (Fig. 1). This is consistent with Zhang et al. (2020), who emphasized that drip irrigation can improve rhizosphere soil moisture distribution and enhance plant water status.

Moreover, soil electrical conductivity (EC) results showed that MP can effectively reduce EC accumulation in the upper soil layer while promoting downward salt migration, thereby easing rhizosphere salt stress (Table 2). This corresponds with observations by Xie, Jin & Wang (2021), who reported that plastic film mulching with drip irrigation can successfully prevent surface salt accumulation in tomato cultivation.

### Physiological mechanisms: synergistic enhancements in ion homeostasis, photosynthetic performance, and ROS defense

This study indicates that MP treatment promotes preferential ion absorption. Specifically, MP can significantly increase the accumulation of K^+^ and Ca^2^^+^ (3.28% and 0.91%, respectively) and reduce Na^+^ (3.5%), thereby increasing the K^+^/Na^+^ ratio to 0.94 and decreasing the Na^+^/Ca^2^^+^ ratio to 3.84 (Table 3). These differences were statistically significant (*p* < 0.05). These values indicate improved membrane selectivity and electrochemical balance under MP, contributing to better cellular stability under salt stress. Similar improvements in K^+^/Na^+^ ratio have been reported in tomato and rice under salinity, indicating that this mechanism is conserved in different plant species (Li, Wang & Chen, 2021; Sharma, Kapoor & Bhatla, 2022). Moreover, ion balance may also be linked to the regulation of key ion transporters such as HKT1 and SOS1, which are known to mediate Na^+^ exclusion and K^+^ retention under salt stress (Zhu, 2016).

In terms of photosynthetic capacity, net photosynthetic rate (*Pn*: 14.8 µmol CO_2_⋅ m^−^^2^ s^−^^1^), stomatal conductance (*Gs*: 0.622 mol H_2_O⋅ m^−^^2^ s^−^^1^), and transpiration rate (*Tr*: 5.28 mmol H_2_O⋅ m^−^^2^ s^−^^1^) of MP is close to CK, reflecting efficient CO_2_ assimilation and stomatal regulation. In addition, intercellular CO_2_ concentration (*C*_*i*_) was relatively low (251 µmol mol^−^^1^), indicating high carbon assimilation efficiency (Fig. 7). These observations are consistent with Chaves, Flexas & Pinheiro (2009), who believed that appropriate water and nutrient strategies can alleviate stress-induced inhibition of photosynthetic apparatus.

For antioxidant defenses, MP treatment can significantly enhance the activities of four key enzymes: SOD, POD, CAT, and APX. SOD and CAT activities reached 158.19 and 20.33 U g^−1^ FW, respectively, far exceeding those under FI (Figs. 5A, 5C). At the same time, MP reduced ROS levels (O_2_^−^ and H_2_O_2_), electrolyte leakage, and MDA content (Figs. 4A–4D). These trends indicate that MP cannot only limit ROS generation, but also enhance the detoxification capacity of plants, effectively mitigating lipid peroxidation. Such enhancements may be driven by transcriptional upregulation of antioxidant enzyme genes and hormonal signaling pathways (such as ABA and ethylene), which are often activated under salt stress (Venkataraman et al., 2021).

These results align with the findings of Munns & Tester (2008), who emphasized that salt-induced oxidative damage can be minimized by efficient nutrient and water delivery systems, particularly under integrated water-fertilizer regimes.

### Innovation: systemic mechanistic framework for water-fertilizer coordination

**Figure 9:**
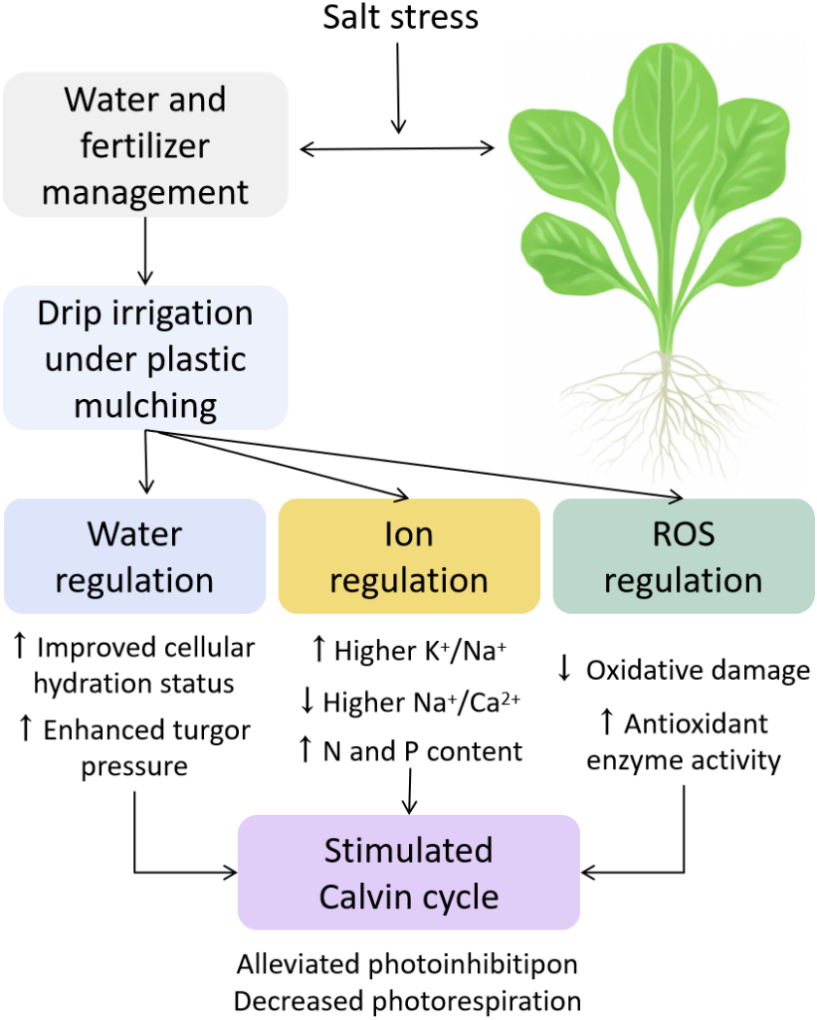
Integrated mechanistic model of water regulation, ion homeostasis, ROS defense, and photosynthetic optimization in pakchoi under salt stress.

The main innovation lies in its factorial design, which combines irrigation modes (I, T, P) and fertilizer types (F, M, C) into nine distinct treatments. This allows for a analysis of phenotypic, physiological, metabolic, and photosynthetic traits under salt stress, and construct an integrated model describing water regulation ion homeostasis ROS defense photosynthetic optimization (Fig. 9). Most previous studies have focused on singular mechanisms such as irrigation or fertilization alone, our approach provides a multi-level, systems-based framework for understanding plant resilience under abiotic stress. As (Zhu, 2016) emphasized, salt tolerance mechanisms should be explored in various physiological layers. The sequential investigation from water potential to ion transport, ROS detoxification, and biomass production reflected this model-based paradigm.

Osmoregulatory compounds such as proline, soluble sugars, and fructose were significantly reduced under MP and CP treatments (Figs. 3A, 3C, 3E), indicating that under reduced stress, plants no longer require excessive energy expenditure for osmoprotection. Alternatively, the decrease in proline and sugars may also reflect a redistribution of metabolic resources from stress defense to growth and reproduction. This reflects higher energy efficiency and physiological stability, and is consistent with the “low-cost stress tolerance” model (Ashraf & Foolad, 2007).

### Prospects

This study demonstrates that coordinated management of irrigation and fertilization under saline conditions can effectively alleviate osmotic stress, ion toxicity, and oxidative damage in pakchoi, thereby improving photosynthetic efficiency and biomass accumulation. Future research should apply multi-omics approaches such as transcriptomics, proteomics, and metabolomics to dissect how MP treatment modulates signaling pathways for water sensing, selective K^+^/Na^+^ transport, ROS scavenging, and ABA-dependent responses at the molecular level, thereby shifting from physiological to mechanistic understanding (Zhang et al., 2022). Specifically, transcriptomics can identify regulatory genes associated with salt-responsive ion transporters, proteomics can quantify antioxidant enzyme profiles, and metabolomics can characterize osmolyte regulation. Moreover, genotypic screening of salt-sensitive pakchoi varieties under MP conditions will help establish a “Genotype-Regulation-Stress” triadic response model, providing precise guidance for crop management in coastal saline lands. In addition, the MP strategy has potential applicability to other crops such as tomato, rice, and wheat grown on saline or sodic soils, broadening its generalizability.

This study used a greenhouse pot experiment without drainage holes under salt stress to simulate the typical coastal saline soil environment with restricted drainage and salt accumulation. However, this experimental setup cannot fully replicate the conditions of well-drained field environments. To improve practical applicability, future studies should scale up from controlled conditions to field trials in varying salinity gradients, water qualities, and climate zones to assess the robustness and scalability of the MP strategy. The integration of phenomics platforms, remote sensing, and machine-learning-assisted irrigation scheduling can further facilitate intelligent water-fertilizer regulation models (Sun, Zhang & Liu, 2022). Future research should address combined abiotic stress scenarios (such as salinity-drought, salinity-heat) to test broad resilience of MP treatment and expand its application, ensuring sustainable leafy vegetable production under climate change conditions.

## Conclusion

This study systematically evaluated the effects of different water-fertilizer management strategies on the growth performance, physiological traits, and antioxidant mechanisms of Pakchoi under salt stress, with a focus on MP treatment (mulched drip irrigation combined with mixed fertilizer).

The findings showed that water-fertilizer regimes can significantly influence soil salinity distribution and ion balance. Treatments with mulched drip irrigation can reduce surface salt accumulation and promote downward migration, and MP treatment can significantly improve K^+^ and Ca^2+^ uptake and reduce Na^+^ content, thereby enhancing ionic homeostasis and salt tolerance.

MP treatment can also effectively alleviate oxidative stress. Low MDA levels and electrolyte leakage, as well as high activities of antioxidant enzymes such as SOD, CAT, POD, and APX, indicating strong ROS detoxification and improved membrane stability.

In addition, water status, photosynthetic performance, and biomass were significantly improved under MP treatment. Improving leaf water potential, maintaining chlorophyll content, and efficient gas exchange support high carbon assimilation and growth under saline conditions. The yield of MP-treated plants was consistently greater than that of other treatments, confirming its optimal productivity.

The MP strategy alleviates salt stress by synergistically optimizing soil salinity profiles, improving ion selectivity, enhancing antioxidant defenses, and improving photosynthetic capacity and water relations. This improvement highlights MP as the most effective water-fertilizer management model for pakchoi cultivation in coastal saline-alkali soils. It is recommended to apply it more widely, combined with salt tolerant varieties and intelligent irrigation and fertilization technology, to promote sustainable and resilient vegetable production systems in saline alkali areas.

## Supplemental Information

10.7717/peerj.20431/supp-1 Supplemental Information 1 Raw data

## Additional Information and Declarations

### Data Availability

The following information was supplied regarding data availability: The raw measurements are available in the Supplementary File.
